# Ecology of conflict: marine food supply affects human-wildlife interactions on land

**DOI:** 10.1038/srep25936

**Published:** 2016-05-17

**Authors:** Kyle A. Artelle, Sean C. Anderson, John D. Reynolds, Andrew B. Cooper, Paul C. Paquet, Chris T. Darimont

**Affiliations:** 1 Earth to Ocean Research Group, Department of Biological Sciences, Simon Fraser University, 8888 University Drive, Burnaby, British Columbia, Canada, V5A 1S6; 2 Raincoast Conservation Foundation, PO Box 2429, Sidney, British Columbia, Canada, V8L 3Y3; 3 Hakai Institute, PO Box 309, Heriot Bay, British Columbia, Canada, V0P 1H0; 4 School of Resource and Environmental Management, Simon Fraser University, 8888 University Drive, Burnaby, British Columbia, Canada, V5A 1S6; 5 Department of Geography, University of Victoria, PO Box 1700 STN CSC, Victoria, British Columbia, Canada, V8W 2Y2

## Abstract

Human-wildlife conflicts impose considerable costs to people and wildlife worldwide. Most research focuses on proximate causes, offering limited generalizable understanding of ultimate drivers. We tested three competing hypotheses (problem individuals, regional population saturation, limited food supply) that relate to underlying processes of human-grizzly bear (*Ursus arctos horribilis)* conflict, using data from British Columbia, Canada, between 1960–2014. We found most support for the limited food supply hypothesis: in bear populations that feed on spawning salmon (*Oncorhynchus* spp.), the annual number of bears/km^2^ killed due to conflicts with humans increased by an average of 20% (6–32% [95% CI]) for each 50% decrease in annual salmon biomass. Furthermore, we found that across all bear populations (with or without access to salmon), 81% of attacks on humans and 82% of conflict kills occurred after the approximate onset of hyperphagia (July 1^st^), a period of intense caloric demand. Contrary to practices by many management agencies, conflict frequency was not reduced by hunting or removal of problem individuals. Our finding that a marine resource affects terrestrial conflict suggests that evidence-based policy for reducing harm to wildlife and humans requires not only insight into ultimate drivers of conflict, but also management that spans ecosystem and jurisdictional boundaries.

Human-wildlife conflicts are widespread, occurring when resource use by human and non-human animals overlap. Interactions can endanger the safety and well-being of humans and wildlife alike, lead to economic loss, and affect the conservation of species by negatively altering public perceptions^1^,^2^,^3^,^4^,^5^,^6^. Animals typically avoid humans^7^, raising the question of what ultimately causes conflicts to occur when and where they do. We propose that investigating the broader ecological context of conflict (hereafter the ‘ecology of conflict’) might help to explain variation in conflict patterns, leading to a better mechanistic understanding and improved prediction and management^8^,^9^,^10^.

We conceive of conflict as a process emerging from proximate and ultimate drivers. Research usually focuses on the former, including human group sizes and behaviours, attractant management, and behaviour of humans and wildlife involved (but see^6^,^8^,^11^,^12^,^13^). Proximate inquiry provides important insights for understanding specific conflicts and how to avoid them^14^,^15^,^16^, but renders limited insight into the timing, location, and causes of broader conflict patterns. Moreover, proximate investigations rarely yield insights generalizable across taxa.

Herein we use a generalizable, ecological approach to explore patterns of human-wildlife conflict, assessing three potential hypotheses of ultimate drivers of conflict. The **‘Problem Individuals’ hypothesis** posits that conflict frequency is driven by the number of conflict-prone (risk-tolerant/bold) individuals in populations^12^,^17^,^18^, and predicts that removing such individuals should reduce subsequent conflict^12^. Consistent with hunger-mediated risk-taking observed across taxa^19^,^20^,^21^,^22^, the **‘Regional Population Saturation’ hypothesis** posits that conflict patterns are driven by wildlife populations exceeding regional carrying capacities, causing nutritionally stressed individuals to take increased risks, leading to increased conflicts with humans. This hypothesis predicts that population reductions (*e.g.* by increased hunting) should decrease subsequent conflict. Finally, via a similar reduction in per capita food supply (and hunger-mediated pathway), the **‘Food Supply’ hypothesis** posits that conflict patterns are driven by changes in regional food supply. It predicts that periods of high conflict should coincide with shortages of natural foods^23^,^24^,^25^. Although not exhaustive, this list of hypotheses allows comparisons of different ultimate ecological processes that might commonly drive conflict in many systems.

We assessed the extent to which conflict between humans and grizzly bears (*Ursus arctos horribilis)* in British Columbia (BC), Canada, might be explained by the three proposed ultimate drivers of conflict. Considerable inter-annual variation exists in patterns of conflict, represented here by human injury and death from, and conflict kills of, grizzly bears from 1960–2014. Similarly, considerable variation exists in patterns of annual human-caused mortality (mostly by hunting^26^), and in annual food availability, especially of spawning Pacific salmon (*Oncorhynchus* spp.), which, in areas where it is available to bear populations, provides a high-caloric and disproportionately important food source to which abundance and fitness are directly related^27^,^28^,^29^. The peak of spawning salmon biomass also coincides with hyperphagia, the pre-hibernation period of intensive energetic demand^30^,^31^,^32^,^33^. Our assessment, which identifies food supply as the hypothesis with most support, illustrates how using a multiple-hypothesis ecology of conflict framework can discriminate among alternate ecological explanations of conflict patterns. Moreover, it illustrates that human-wildlife conflict might be affected by ecological processes that are broadly applicable across space, time, and taxa, and that might extend well beyond administrative and ecological boundaries.

## Results

### Temporal and spatial patterns of conflict

**Figure 1:**
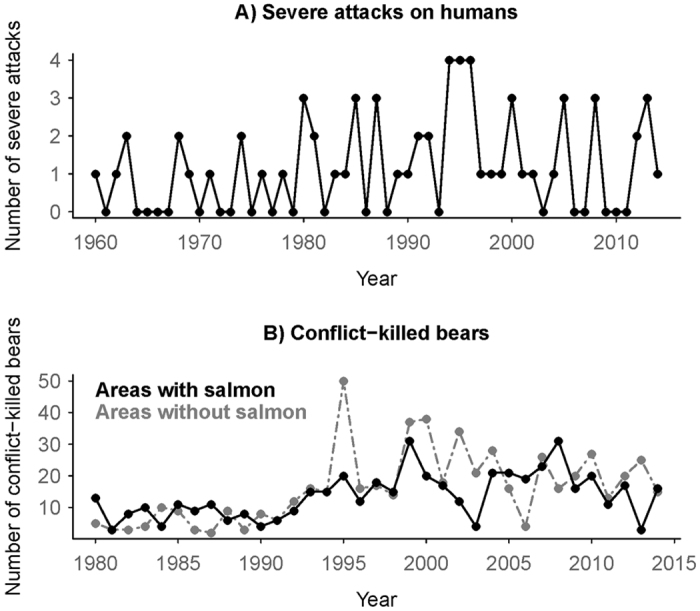
Number of grizzly bear (*Ursus arctos horribilis*)-human conflicts by year in British Columbia, Canada: (**A**) Annual number of severe (causing hospitalization) grizzly bear attacks on humans for the whole province combined, 1960–2014 (top), and (**B**) annual number of conflict-killed grizzly bears in areas with (black lines) and without (grey lines) spawning salmon, 1980–2014.

**Figure 2:**
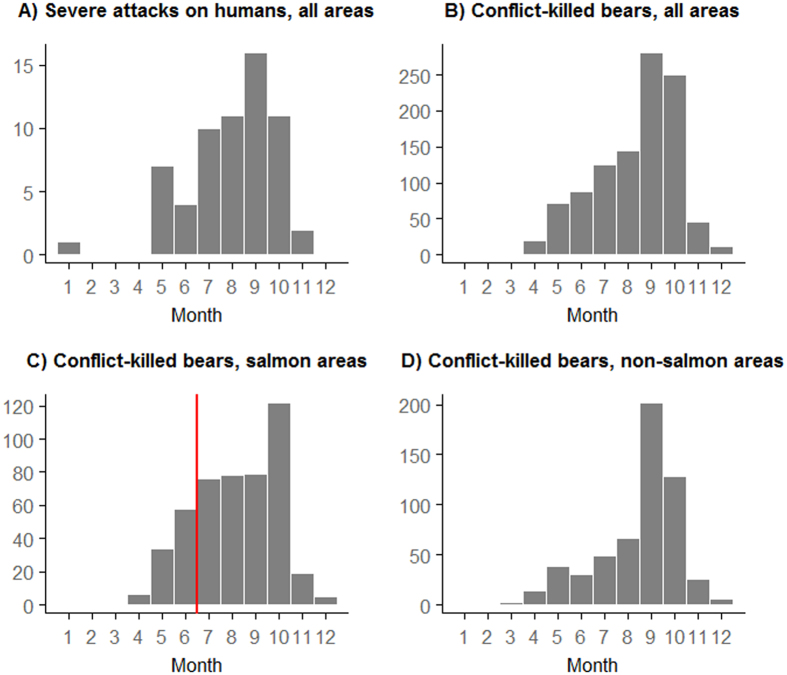
Number of conflicts between grizzly bears (*Ursus arctos horribilis*) and humans by month in British Columbia, Canada: (**A**) monthly number of severe grizzly bear attacks on humans from 1960–2014 (n = 64), monthly number of conflict-killed grizzly bears from 1980–2014 for (**B**) the whole province combined (n = 1145), (**C**) for areas with salmon (n = 546), and (**D**) those without salmon (n = 599). Red vertical line indicates approximate onset of most salmon runs.

**Figure 3:**
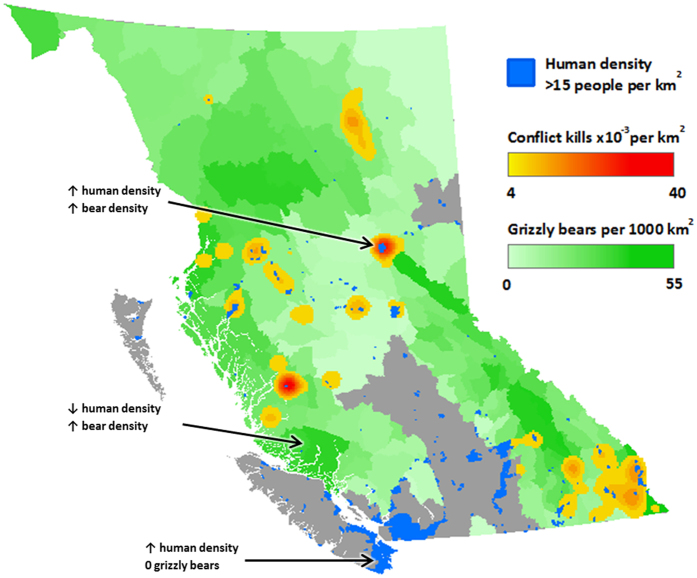
Hotspots of grizzly bear (*Ursus arctos horribilis*) conflict kills in British Columbia, Canada, 1978–2014. Increasingly ‘hot’ colours (yellow to red) represent an increasing density of conflict kills across the study period. Darker green areas have higher estimated grizzly population densities (grey areas have no grizzly bears). Blue areas are those with a human density ≥15 people per km^2^. Generated with ArcMap 10.2, www.esri.com.

Between 1960–2014, severe attacks on humans were rare (mean of 1.18/year). Although their frequency increased slightly through time, attacks were episodic, with considerable inter-annual variation (from 0–4/year; Fig. 1A). Most (50 of 62; 81%) occurred late in the year (from July-onwards; Fig. 2A). Similarly, between 1980–2014 conflict kills of grizzly bears (involved in human-wildlife conflict and killed as a result, either by private citizens or provincial agents) were episodic, increasing somewhat over time (Fig. 1B), and primarily occurred late in the year, peaking in autumn (857 of 1042, 82% occurred from July-onwards; Fig. 2B). This seasonal pattern was consistent in areas with and without spawning salmon (379 of 479, 79% occurred from July-onwards in areas with salmon, Fig. 2C; 478 of 563, 85% occurred from July-onwards in areas without salmon, Fig. 2D). Conflict-killed bears were typically younger than hunter-killed bears (median age of 2 and 5 years, respectively; Supplementary Fig. 1), and occurred closer to towns (median distance of 17 km for conflict-killed and 44 km for hunter-killed; Supplementary Fig. 1). Conflict kills were clustered in hotspots, mostly where high estimated densities of grizzly bears overlapped with human habitation (Fig. 3).

### Predictors of conflict kills

We used model-averaged, hierarchical models to identify associations between patterns of conflict (*i.e.* annual number of conflict-killed bears per km^2^) and spatial and temporal variation in ecological predictors, and to assess the relative support for our three hypotheses (problem individuals, regional population saturation, food supply). Because salmon only spawn in some areas, we fitted two separate model-averaged models: a ‘salmon areas’ model that included temporal variation in salmon availability, limited to areas with spawning salmon, and a ‘full region’ model that included the full province but excluded salmon availability as a predictor. Both models had reasonable fit to the data (*e.g.* Supplementary Fig. 2).

**Figure 4:**
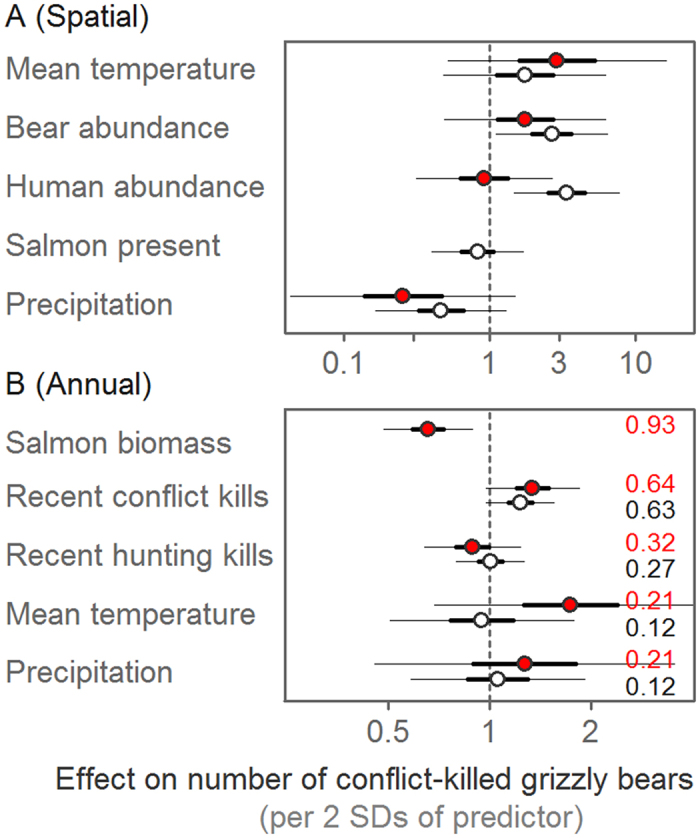
Effect of ecological variables on annual number of conflict-killed grizzly bears (*Ursus arctos horribilis)* in British Columbia, Canada, 1980–2013. Dots represent centred (mean-subtracted) and scaled (divided by 2 SD) parameter estimates and thick and thin bars represent the 50% and 95% confidence intervals, respectively, from salmon areas (red, with filled circles) and full region (black, with hollow circles) models. Relative variable importance values are shown in panel B. Results shown are model-averaged across candidate model sets (Supplementary Tables 1 and 2).

Spatial predictors accounted for considerable regional differences in conflict patterns in both models (Fig. 4A). Areas with larger estimated bear densities had more conflict in the full region model, as did areas with larger human densities. We did not find an effect of spatial differences in precipitation and temperatures on conflict in either model. Similarly, we did not detect a difference in conflict prevalence between areas with and without salmon.

Temporal variables revealed support only for the food supply hypothesis. Contrary to the problem individuals hypothesis, we did not find that previous conflict kills were associated with subsequent conflict levels, with 95% confidence intervals of coefficients that overlapped zero, and with moderate relative variable importance (‘RVI’; 0.63 for full region and 0.64 for salmon areas model; Fig. 4B, Supplementary Table 1). Contrary to the regional population saturation hypothesis, we found little evidence of an effect of previous hunting levels on conflict, and low RVI (0.27 for full region and 0.32 for salmon areas; Fig. 4B, Supplementary Table 1). Although we found little evidence of an effect of terrestrial food supply on conflict, with 95% confidence intervals of coefficients for annual climatic proxies for terrestrial food availability (temperature and precipitation) that overlapped zero, and low RVI (0.12 for full region and 0.21 for salmon areas; Fig. 4B), we found the most support for marine-derived food supply affecting conflict: annual variation in salmon biomass had the highest RVI of all annual variables in the salmon areas model (0.93, Fig. 4B, Supplementary Table 1). Years with lower salmon abundance were associated with increased conflict (Fig. 4B, Supplementary Table 1). For example, conflict increased by 20% (6%–32% [95% CI]) for each 50% decrease in the geometric mean of salmon biomass for a grizzly bear population with average salmon variability (Supplementary Fig. 3).

## Discussion

### Discriminating among hypotheses

Of the three hypotheses assessed, we found most support for the food supply hypothesis. Contrary to the predictions of the problem individuals hypothesis, we did not find a reduction, but instead a suggestive trend of an *increase,* in conflict kills following periods with high conflict removal of bears. Apparent increases in conflict following increased kills might be explained by a number of mechanisms, including, but not limited to, social effects on the hunted populations (*e.g.*^6^,^34^), reduced tolerance of humans towards wildlife following recent periods of conflict, or the presence of persistent anthropogenic attractants across multiple years. However, in this particular case the suggestive trend was not substantiated statistically, and hence did not constitute evidence of any discernible effect of conflict kills on subsequent conflict. That we did not detect an effect is perhaps not surprising because most conflict involved younger individuals^35^. If conflict-proneness decreases with age, then removing young individuals might not reduce future conflict because individuals would become less conflict-prone as they age, with or without management intervention.

Similarly, we did not find support for the regional population saturation hypothesis. Whereas areas with higher estimated densities of grizzly bears and humans experienced more conflict, annual hunting intensity had no measurable effect on subsequent conflict, suggesting attempted population reduction via hunting might not be effective in mitigating conflict (see also^24^,^36^,^37^). Moreover, as in other wildlife systems where hunting is used in part to mitigate conflict^34^, individuals killed by hunters differed from those typically involved in conflict: in our system, hunter-killed bears were older and lived farther from human habitation.

We found the most support for the food supply hypothesis, with salmon availability being the annual variable with the greatest measured importance for explaining conflict prevalence. However, the use of coarse measures might have obscured other effects related to food supply. For example, estimated spawning abundance is a crude proxy for salmon availability^38^,^39^. In addition, whereas we did not find a strong association with annual climate measures (temperature and precipitation), these measures might have limitations as proxies for terrestrial food availability. Whereas changes in climate and weather would be expected to affect terrestrial food availability, responses among plant species vary considerably^40^, and weather-related events such as late frosts (*e.g.*^40^) or acute weather events might have an effect not detectable with the available data. Moreover, given the size and bioclimatic diversity of British Columbia, a generalizable effect of climate on food availability might not be realistic across this large and varied region. We suggest that monitoring of terrestrial food availability, at least where possible at finer scales, might help to elucidate food-related mechanisms further and provide considerable improvements over the proxies we used. Despite not detecting a relationship with terrestrial foods, most conflict kills in all areas occurred in the latter part of the year and peaked in autumn, coincident with hyperphagia. This suggests that additional food-related causation might be important, even in areas without salmon (*e.g.*^25^,^37^). Although attacks were too rare to model, they similarly peaked during hyperphagia, additionally suggesting food-related causation. Although the particularities of hyperphagia’s effects on human-bear conflict are not generalizable to all conflict systems, it provides an illustrative example of how the timing of resource need, resource availability, and human-wildlife conflict might offer insight into potential ecological associations.

### Management considerations

Our findings suggest that reconsidering lethal removals and hunting, approaches commonly prescribed by management to reduce conflict^4^,^6^,^34^,^41^ might be warranted. Removal of individuals might be considered necessary in some circumstances, such as when individuals exhibit predatory behaviour towards humans^13^,^24^,^42^, or when specific individuals are involved in repeated livestock predation^3^,^34^. However, as we observed, increasing overall rates of removal might not affect subsequent rates of conflict. Improved conflict management might instead include addressing underlying ecological stressors, such as protecting or restoring natural food (*e.g.* from overharvest or habitat destruction). Additionally, a focus on understanding the underlying ecology of conflicts could focus limited resources on mitigation efforts (including education and attractant management) when and where conflicts are most likely to occur. Predicting conflicts could enable a proactive, non-lethal approach to prevention, reducing the impetus for the reactive, often lethal responses that might offer only limited benefit in the long term.

Broadly, management conducted without consideration of underlying ecology could lead to errors, and in some instances, harm. For example, in cases where increases in conflicts are driven by reduced food supply but are assumed to be caused by increasing wildlife population densities (*i.e.* regional population saturation hypothesis), managers might fail to address the underlying issue and instead subject populations already facing stress and potential declines to increased lethal control or hunting.

Moreover, in many jurisdictions worldwide, including BC, wildlife populations (and the processes that affect them) transcend ecological and/or jurisdictional boundaries, yet are managed by agencies that do not^43^,^44^. This ecological mismatch limits the ability of agencies to address important ecological drivers of conflict like those detected here. For example, in BC, grizzly bears (and human-bear conflict) are managed by the provincial government of British Columbia, whereas the spawning salmon on which many populations depend are managed by Fisheries and Oceans Canada. The provincial government has the ability to destroy grizzly bears, but not to manage their food, whereas our results suggest the former might be less effective than the latter. Additionally, whereas the importance of nutrient subsidies in ecology is well-studied, including in this region (*e.g.*^33^,^43^,^45^,^46^), to our knowledge it has never before been assessed as a driver of human-wildlife conflict. Effective prevention and mitigation of human-conflict might require agencies to manage at more ecologically relevant scales, and manage not only conflict-implicated species, but also the foods on which they rely. Similarly, encouraging agencies responsible for prey species (*e.g.* salmon) management to also consider dependent communities of wildlife consumers might help to mitigate the current disconnect. Canada’s ‘Wild Salmon Policy’, which requires ecosystem considerations in fisheries allocations^47^, provides an example of a potential mechanism, though it has yet to be implemented^48^. Applying this policy for mitigating human-wildlife conflict might provide a tractable test case for cross-biome ecosystem management while benefiting both ecosystem conservation and human safety.

### Ecology of conflict

Our study illustrates a generalizable multiple-hypothesis-testing approach for assessing ultimate ecological drivers of human-wildlife conflict. Instead of presenting a single hypothesis for observed patterns, we concurrently weighed support for multiple hypotheses within a single system^49^. This approach not only provides greater confidence in the associations detected, but also is amenable to various taxa, geographies, and ecological contexts. For example, whereas food supply seemed to have the greatest impact on conflict patterns in our study system, additional mechanisms, including but not limited to our alternate hypotheses, might be at play here or elsewhere, might interact with one another, and might be context-dependent. Applying this approach broadly might help to increase the understanding of ultimate drivers of human-wildlife conflict in any system, while identifying commonalities among human-wildlife conflict systems worldwide.

## Methods

We assessed patterns in timing, location, and age of grizzly bears involved in human-wildlife conflict, represented by conflict kills of bears and attacks on humans in British Columbia (BC), Canada. We modeled the association between annual variation in ecological variables and human-bear conflict frequency. Whereas we were primarily interested in inter-annual patterns for assessing the relative support for our three hypotheses, we included spatial variables in our model to account for ecological differences among populations.

All analyses were performed at the scale of Grizzly Bear Population Unit (hereafter ‘population’), which is designated by the provincial government of BC and is thought to correspond with geographically and genetically relevant sub-populations (50; Supplementary Methods). The ‘habitable area’ (area excluding glaciers and water bodies) of these populations ranges from 2,698 km^2^ to 49,268 km^2^ (mean = 13,316 km^2^; Supplementary Fig. 4).

### Conflict kills

We compiled the annual number of conflict-killed grizzly bears from the ‘Compulsory Inspection Database’ (hereafter ‘CID’), which contains the date, location, and cause (*e.g.* hunt, conflict kill, road accident) of all known human-caused grizzly bear deaths in BC, from 1977–2014^50^; Supplementary Methods; Supplementary Fig. 5). We also extracted age estimates from the database, which were available for 75% of human-caused kills. We used entries only from 1980-onwards because data quality improved considerably after this point (T. Hamilton pers. comm.). We excluded conflict kills from areas where grizzly bears are considered extirpated or threatened (n = 37; Supplementary Methods) because such kills are anomalies, whereas we were interested in generalizable patterns. We included only late-season conflict (occurring from July 1st onwards; 79% of recorded conflict kills; Fig. 2) as a response in our models, because abundance of spawning salmon (a predictor in our model) and bear predation on salmon peak from summer through autumn^51^. We used the ArcGIS^52^ spatial analyst kernel density estimator, which uses a quadratic kernel function as described in^53^ to visualize the spatial density (number per km^2^) of conflict kills.

### Attacks

We combined a database of all known dates and locations of attacks in BC from 1960 to 1997 provided by Stephen Herrero^54^ with a database of attacks from 1998 to 2014 provided by the BC Ministry of Environment. We included only ‘severe’ attacks for both time periods (for which details on hospitalization differed: pre-1998 severe attacks included fatalities, and injuries requiring >24 hours of hospitalization, whereas from 1998 onwards, when data on hospitalization duration were absent, severe attacks included fatalities, and injuries of a severity requiring hospitalization [*e.g.* dismemberment and broken bones]) when examining trends through time. We did this because all severe attacks are recorded by the provincial government, whereas the proportion of ‘minor’ attacks (those not requiring medical attention) recorded is unknown (but see Supplementary Fig. 6 for timing of all recorded attacks).

### Spatial correlates

We accounted for geographic variation in climate (temperature and precipitation), grizzly bear and human population densities, and presence or absence of spawning salmon. To assess climatic differences among populations we used the program ClimateBC^55^, which downscales climatic variables obtained from weather stations across the province to an 800 m × 800 m resolution with high accuracy (R^2^ >> 0.9 between most predicted and weather-station measurements^55^,^56^). We created a 4 km × 4 km grid of points across each population’s habitable area (Supplementary Fig. 4) and calculated the log-transformed mean of climate normals (mean spring and summer temperatures and log total spring and summer precipitation from 1981–2010) extracted at each point. To assess differences in grizzly bear densities among populations, we divided 2012 population estimates^50^ by habitable area in each population and log-transformed the quotient. We used the 2011 Canadian census^57^ for spatial assessments of human densities. We attributed human population counts from the finest spatial scale available (census subdivisions) to each bear population unit based on percent overlap of the two spatial scales, divided by habitable area of the bear population unit. For a bear population unit with k subdivisions the calculation was:


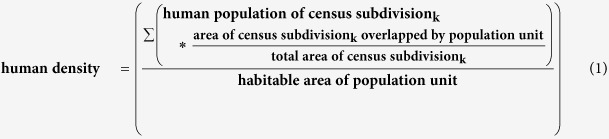


We used a province-wide database of spawning salmon enumerations^58^ to attribute presence/absence of spawning salmon to each population.

### Annual correlates

Within each population, we assessed inter-annual variation in number of recent conflict kills, number of recent hunting kills, mean spring and summer temperature and precipitation, and annual salmon availability to evaluate the relative support for our three hypotheses (problem individuals, regional population saturation, food supply). We used climate as a coarse but broadly applicable proxy for terrestrial bear food availability because estimates of terrestrial food across BC do not exist, though climate has been linked to food availability and human-wildlife conflict elsewhere^59^. Specifically, given their broad association with net productivity^60^, we used measures of temperature and precipitation, during the growing season (spring and summer) of vegetative grizzly bear foods, including shoots, sedges, and berries^31^,^40^,^61^,^62^. We calculated annual values of mean spring and summer temperature and log-transformed total precipitation from ClimateBC values extracted from a 4 km × 4 km grid of points across each population. We calculated the number of hunting and conflict kills in recent years using a 3-year rolling window, *e.g.*:


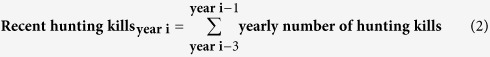


Within each population, recent hunt and conflict kills were scaled by 2 standard deviations with the mean subtracted, providing a measure of inter-annual variation scaled to the magnitude and variability of these measures in each population. We did not include human population as an annual predictor because such data do not exist annually, and there was little temporal variation among censuses in this period. Similarly, we did not include inter-annual variation in grizzly bear densities because such data do not exist in BC^26^. We assessed inter-annual variation in spawning salmon biomass across the province from 1980 to 2013, using the Fisheries and Oceans Canada nuSEDS database (Supplementary Methods^58^; see^63^,^64^ for caveats). At each salmon count location (Supplementary Fig. 7), we calculated the annual biomass of each species individually, omitting species-stream time series with data missing for more than eight years total, or for three or more consecutive years. We estimated missing counts in the remaining time series by multiple imputation with a Ricker-logistic model fitted to each stream and species (Supplementary Methods; Supplementary Fig 8). We attributed each stream salmon count location to a bear population unit and calculated the total annual salmon biomass for each population as the geometric mean of annual stream biomasses of all salmon species combined (Supplementary Methods). Within each bear population, annual biomass was scaled by 2 standard deviations with the mean subtracted, providing a measure of inter-annual variation scaled to the abundance and variability of salmon in each population.

### Analyses

We assessed associations between ecological correlates and conflict patterns using the R^65^ package glmmADMB, which estimates parameters by maximizing likelihood^66^. We used a hierarchical modeling approach combining variables that vary spatially among populations with those that vary temporally within each population (Equation 3). We centred and scaled all predictors (subtracted the mean from each observation and divided by 2 standard deviations) to facilitate meaningful comparisons of effect sizes among predictors^67^. We ran ‘full region’ models that included all populations, and ‘salmon areas’ models restricted to populations with estimated salmon availability. We visually inspected residuals plotted against each predictor and the fitted values and did not detect any remaining strong patterns. Similarly, we visually assessed autocorrelation in residuals and observed little spatial autocorrelation, and substantial temporal autocorrelation in only one population (excluding this population had no qualitative effect on our results), so we did not include autocorrelation terms for model simplicity.

We modelled the number of conflict-killed bears (*y*) per unit area (km^2^) in year *i* and population unit *j* as


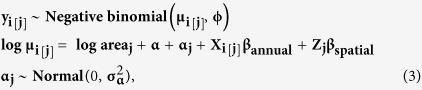


where μ_i[j]_ and *ϕ* represent the mean and size parameters of the ‘NB2’ parameterization of the negative binomial distribution^68^ in glmmADBM, which was used because our data were over-dispersed (*ϕ* in fitted model estimated as 0.72 for salmon areas and 0.62 for full region model); *X*_i[j]_ represents a vector of annual predictors (salmon biomass, recent conflict kills, recent hunt kills, mean spring and summer temperature, total spring and summer precipitation) with associated *β*_annual_ coefficients; and *Z*_j_ represents a vector of spatial predictors (grizzly population density, human population density, mean spring and summer temperature, mean total spring and summer precipitation, salmon present [yes/no]) with associated *β*_spatial_ coefficients. The α_j_ term represents random deviations from the overall intercept *α* that vary with population unit and have variance 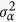. The term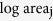 is an offset term representing the habitable area of each population.

We used an information theoretic approach^69^, whereby we assessed relative variable importance to weigh support for our hypotheses, and conducted model averaging across two sets of candidate models (‘full region’ and ‘salmon areas’; Supplementary Table 2). Spatial predictors were used to account for spatial differences in conflict patterns and allow for inference about our temporal hypotheses. We assessed support for our three hypotheses using coefficient estimates and variable importance of temporal predictors in the two model-averaged models. For the problem individuals hypothesis we assessed previous 3 years of conflict kills, for the regional population saturation hypothesis we assessed previous 3 years of hunting kills, and for the food supply hypothesis we assessed marine-derived food using salmon availability (salmon areas model only), and terrestrial food using climatic variables (annual precipitation and mean temperature; both models) combined. All analyses were performed using R 3.1.2^65^ (for code and source data see github.com/kartelle/ecology-of-conflict/).

In describing overall patterns of conflict, we used all data available (spanning 1960 to 2014), whereas we were limited to data from 1980 to 2013 for modeling purposes because the salmon database and climate data we used did not extend beyond 2013, and reliable conflict-killed bear data were only available from 1980-onwards.

## Additional Information

**How to cite this article**: Artelle, K. A. *et al*. Ecology of conflict: marine food supply affects human-wildlife interactions on land. *Sci. Rep.* **6**, 25936; doi: 10.1038/srep25936 (2016).

## Supplementary Material

Supplementary Information
