# Supplementary material for: Ecology of conflict: marine food supply affects human-wildlife interactions on land
Source: Sci Rep. 2016 May 17;6:25936. doi: 10.1038/srep25936 (PMC4869031; doi:10.1038/srep25936)
Supplement: Supplementary Information [file srep25936-s1.pdf]

Supplementary Information for Ecology of conflict: marine food supply affects human-wildlife interactions on land

Kyle A. Artelle, Sean C. Anderson, John D. Reynolds, Andrew B. Cooper, Paul C. Paquet, Chris T. Darimont

Supplementary Methods

Spatial scale

We used the 2012 grizzly bear population units (hereafter 'populations'; [1]; http://catalogue.data.gov.bc.ca/dataset/grizzly-bear-population-units) as our spatial scale of analysis. The publicly-available shapefile depicting these contains some 'donut holes' – areas between polygons not assigned to any population. We deleted and excluded these from calculations of 'habitable area' and other spatial variables. Kills were attributed to nearest population in most cases (see below). Of the 57 populations (as delimited in 2012), 46 were viable throughout 1980-2013, and 21 of these had salmon. Across viable populations, grizzly bear densities ranged from 1.9×10^{-3} to 5.26×10^{-2} bears per km^2, with a mean of 2.54×10^{-2} and sd of 1.22×10^{-2}.

Preparation of database containing human-caused kills of grizzly bears

We compiled conflict and hunt kills of grizzly bears from the Compulsory Inspection Database[2]. For modelling purposes, each kill between 1980-2013 was assigned spatially to a population. Hunt and conflict kills in extirpated (hunt n=17; conflict n=35) and threatened (hunt n=133; conflict n=89) regions were excluded from models because hunting was excluded for at least part of the study period in these areas, likely resulting in dynamics that differ from the rest of the province. There were 217 hunt and 37 conflict kills that were excluded because they lacked spatial information. There were 8 hunt and 17 conflict kills with spatial data that occurred outside the limits of populations. Most of these were due to GIS errors – they were immediately adjacent to populations or in "donut holes" between populations – and were assigned to the nearest population. Four cases of human-caused kills on Vancouver Island (3

conflict kills and 1 hunt) were grouped (and excluded from models) with kills in extirpated regions because grizzly bears are present there only occasionally. All remaining hunt and conflict kills were used for modeling: within viable populations, the annual number human-caused kills from conflicts ranged from 0 to 48 (mean = 6.2, sd = 6.1), and from hunts ranged from 0 to 27 (mean = 0.6, sd = 1.6).

Salmon biomass calculations

The Fisheries and Oceans Canada nuSEDS salmon database [3] comprises spawning enumerations of all five species of Pacific salmon in the province. Salmon biomass in each stream was calculated by multiplying raw counts with average species-specific weights of individuals, assuming a 1:1 sex ratio. Values per fish used, in kilograms, were: Chinook: 13.6, Chum: 5.2, Coho: 3.2, Pink, odd years: 2.4, Pink, even years: 1.7, Sockeye: 2.7 [4].

Data gaps were common in the dataset, with species-specific counts of salmon missing from many streams in many years [5,6]. We excluded from the analyses species-stream times series with counts missing for more than 8 years in total, or more than 3 consecutive years. We imputed missing annual biomass values in remaining species-streams with a Ricker-logistic model fit to the number of salmon counted of each species in each stream:

$$x_t = x_{t-1} + r_{max}\left(1 - \frac{N_{t-1}}{K}\right) + \epsilon_t$$

$$\epsilon_t \sim \mathrm{Normal}(0, \sigma^2),$$

where N_t is the number of salmon counted at year t, $x_t = \ln N_t$, r_{max} represents the theoretical maximum per capita growth rate that is obtained as N_t approaches 0, K represents the carrying capacity, and ϵ_t represents the normally distributed random process noise (the stochastic jumps between time-steps that are not explained by the Ricker-logistic model) with mean 0 and standard deviation σ. Any years with missing values were estimated as additional parameters.

To fit the Ricker-logistic models, we set the prior on K as uniform between zero and the maximum observed abundance (as in [7]). We set the prior on $\mathrm{r_{max}}$ as uniform between 0 and 5. We set a weakly informative prior of Half-Cauchy$(0,2.5)$ [8,9] on the process noise standard deviation.

We fit our imputation models with Stan [10–12]. We initially sampled from our models with 10,000 iterations across 4 chains, discarding the first half of the iterations on each chain as warmup, for a total of 20,000 samples. We checked if $\hat{\mathrm{R}}$ (a measure of chain convergence) was less than 1.05 and if $\mathrm{n_{eff}}$ (the number of effective uncorrelated samples) was greater than 100. For all streams that did not meet these criteria, we resampled from the models with 200,000 iterations (100,000 warmup), 4 chains, and saved every 5th iteration. We then fit the remaining three streams that had not yet converged according to our criteria of $\hat{\mathrm{R}} < 1.05$ and $\mathrm{n_{eff}} > 100$ with 1 million iterations (discarding the first half), 4 chains, and saving every 10th iteration. We also visually inspected the chains of each final model for convergence. We used the median posterior imputed values for our primary analysis.

To test whether our conclusions were sensitive to uncertainty in the posterior distribution, we drew 8 samples from the posterior of imputed values of each time series to run as a multiple imputation sensitivity test (Supplementary Figure 8A). We fit the full model (not the model-averaged model) to each of these 8 replicate datasets to illustrate how the model would perform under alternative plausible imputation scenarios, and found that results did not change qualitatively.

To test how well the Ricker model imputed missing data points we randomly removed one data point (biomass in a given year) from each complete time series (time series of a given species in a given stream without any missing years). Comparing the imputed values from this simulation to actual values revealed that the Ricker model approximated the missing data reasonably well (Supplementary Figure 8B)

When attributing stream salmon counts to bear population units we used the geometric mean of annual stream biomasses to give more weight to smaller streams, given their disproportionate importance for bear predation on salmon [13]. Running the analyses with total biomass instead of geometric mean did not qualitatively change the results.

Supplementary Figures

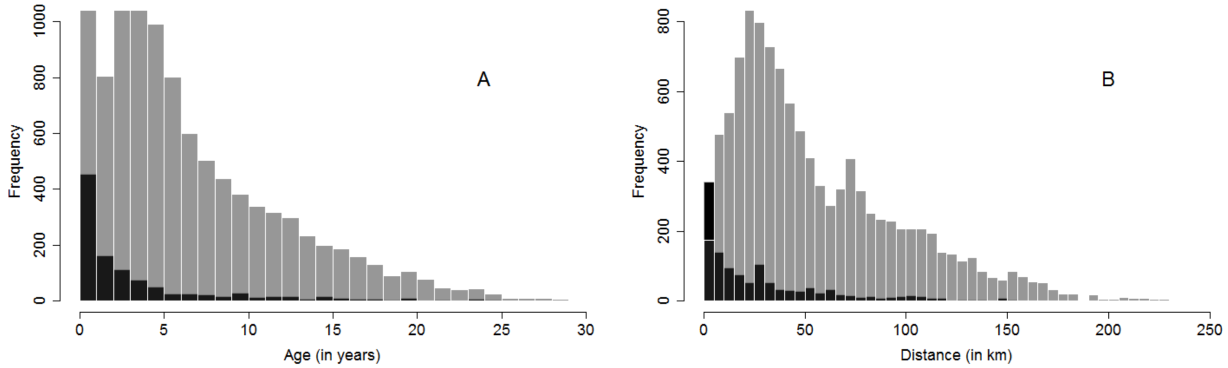

Supplementary Figure 1 A) Age and B) distance from nearest city, town, or community of conflict-killed (black bars) and hunter-killed (grey bars) grizzly bears (*Ursus arctos horribilis*) in British Columbia, Canada, 1978-2014.

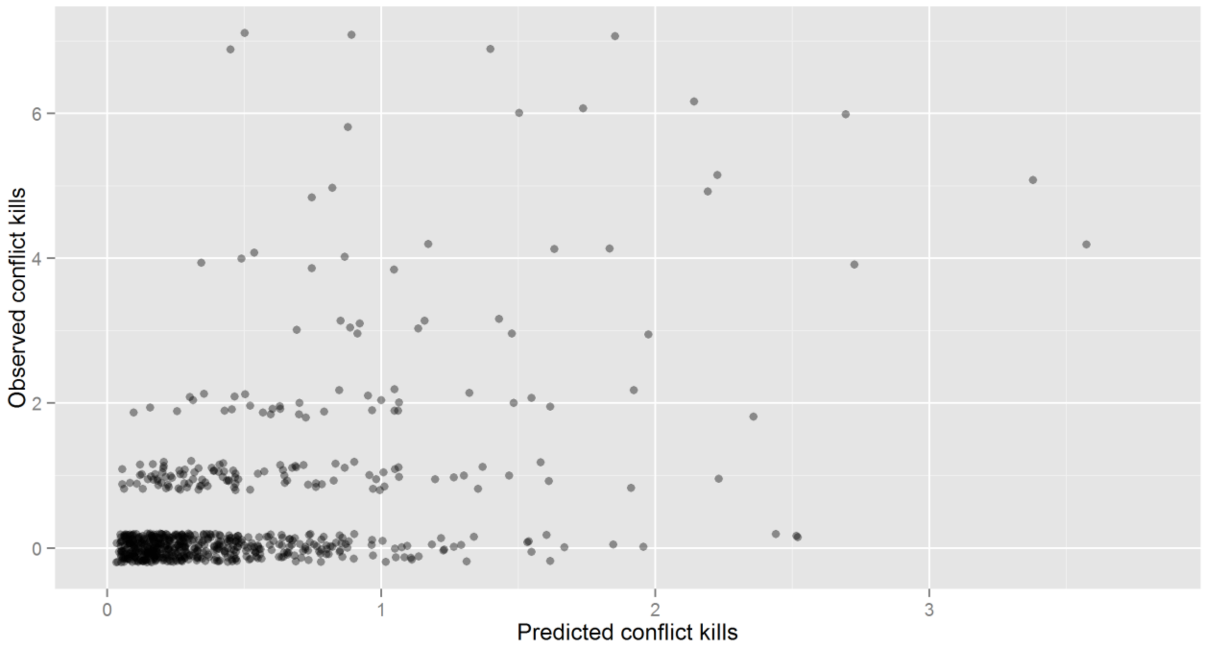

Supplementary Figure 2 Observed versus predicted annual number of conflict-killed grizzly bears (*Ursus arctos horribilis*) in British Columbia, Canada, 1980-2014, from the model-averaged salmon areas model. Predictions shown are based on fixed effects only added to random effect estimates for each grizzly bear population unit. Two points were omitted (predicted conflict kills > 4), and positions of points on y-axis were jittered, to better show the bulk of data.

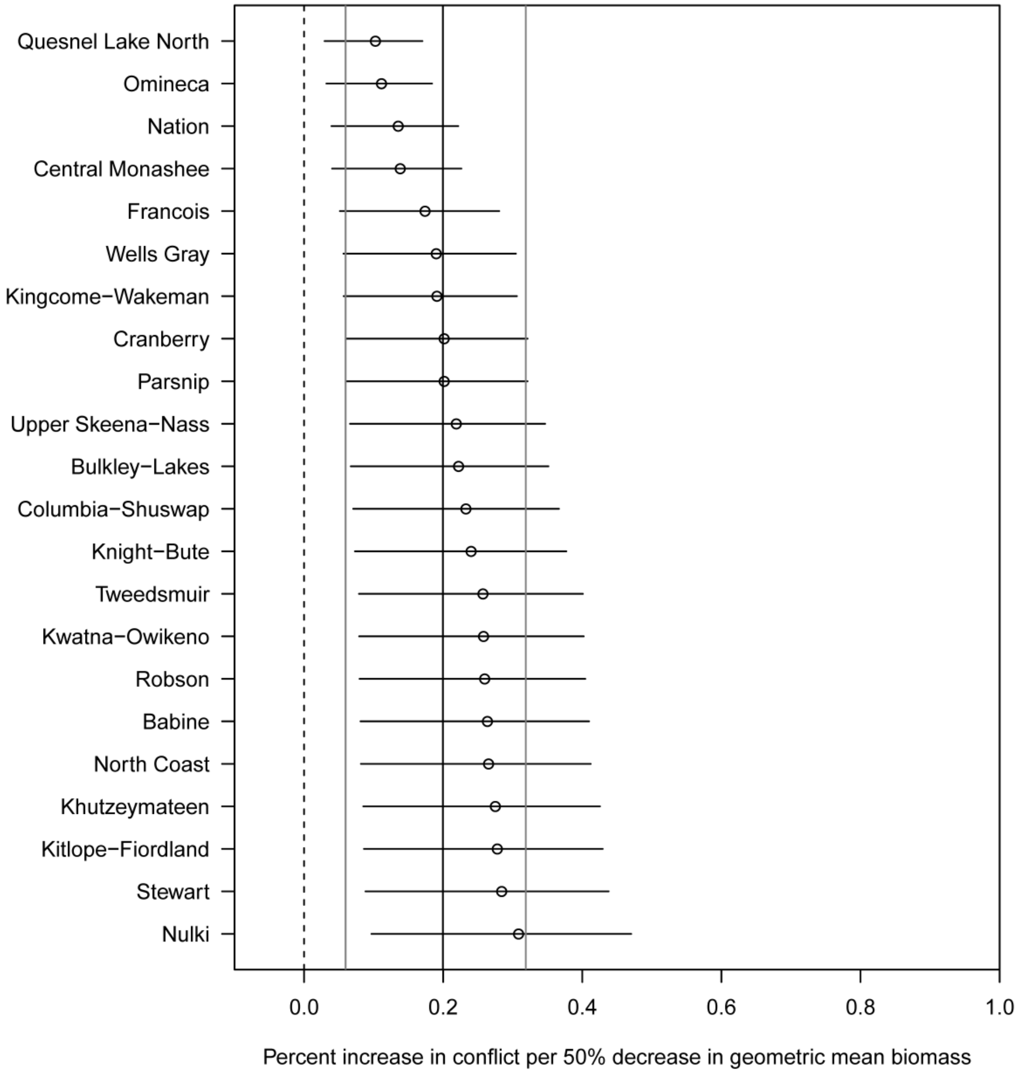

Supplementary Figure 3 Effect of salmon availability on annual number of conflict-killed grizzly bears (*Ursus arctos horribilis)* for each grizzly bear population unit analyzed in British Columbia, Canada, 1980-2013. Open circles represent percent increase in conflict per 2-fold (50%) decrease in geometric mean of salmon biomass and bars represent the 95% confidence intervals. Black solid vertical line and dark grey solid lines indicates the percent increase in conflict-killed bears and 95% CI, respectively, per two-fold (50%) decrease in the geometric mean of salmon biomass for a population with average variability of salmon. The back-transformed effect sizes vary by population because the salmon predictor was scaled within each population.

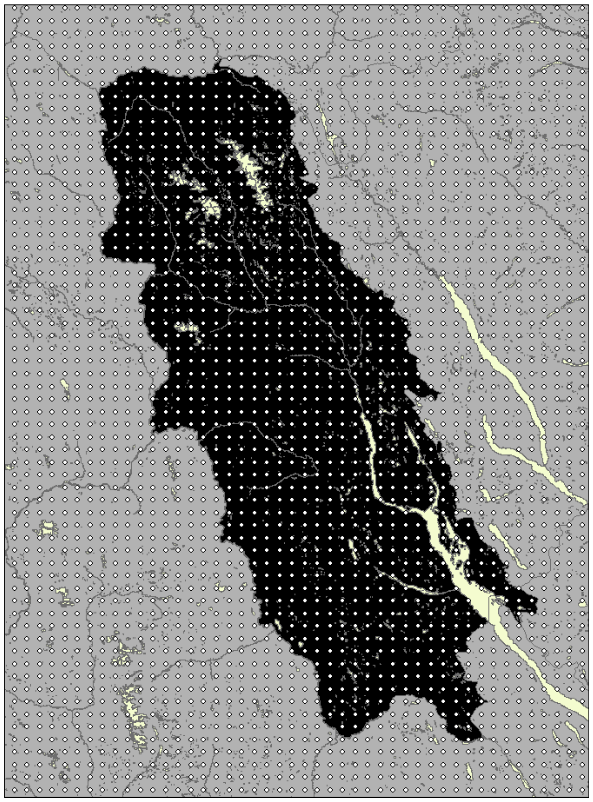

Supplementary Figure 4 Example of approach used to estimate habitable area of a population and to extract population-level climate indices. Habitable area of the Babine grizzly bear (*Ursus arctos horribilis*) population unit is shown in black. Glaciers and waterbodies (lakes and rivers) are shown in white, and excluded from habitable area. Each dot represents a location from which climate normals (typical temperature and precipitation from 1981-2010) were extracted. Generated with ArcMap 10.2, www.esri.com.

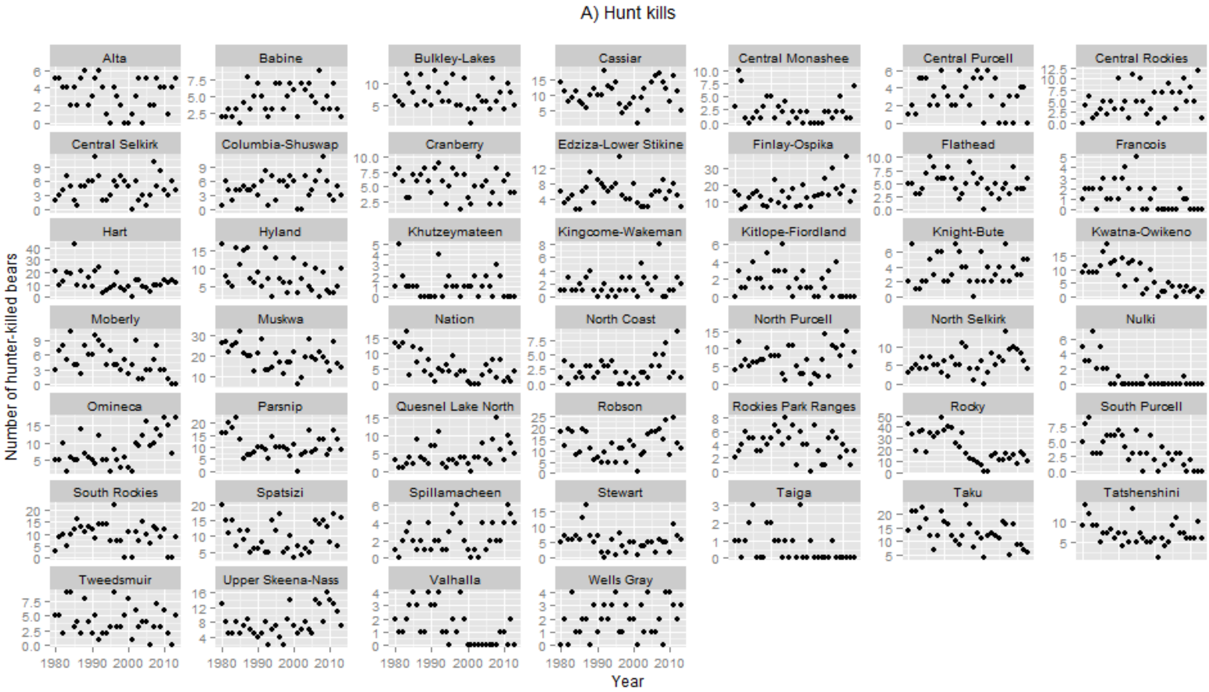

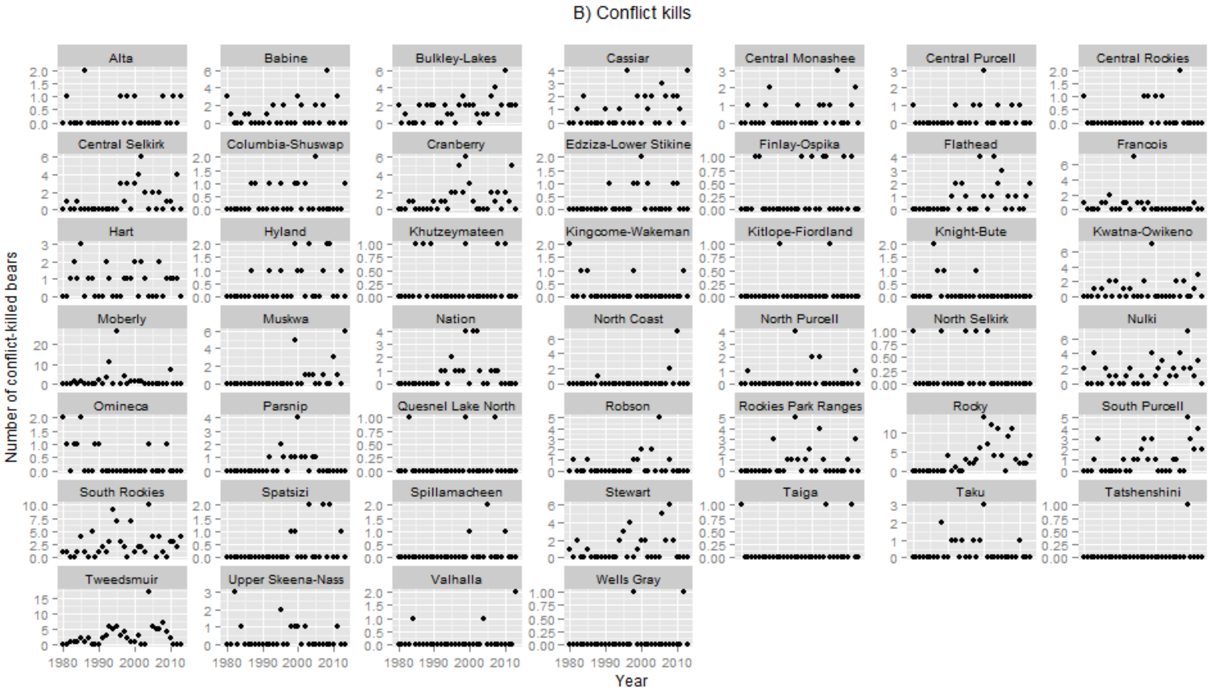

Supplementary Figure 5 Number of A) hunter-killed and B) conflict-killed grizzly bear (*Ursus arctos horribilis*) by year in each Grizzly Bear Population Unit in British Columbia, Canada, used to model grizzly bear-human conflict from 1980-2013.

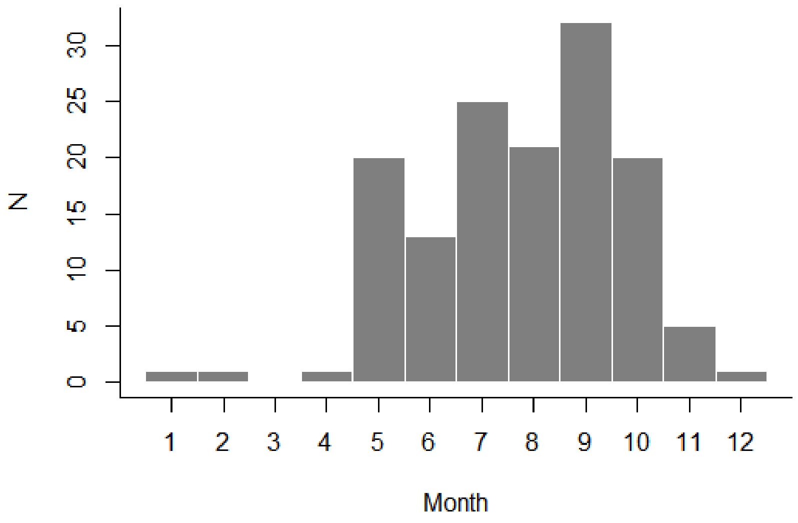

Supplementary Figure 6 Number of grizzly bear (*Ursus arctos horribilis*) attacks (including attacks that did not cause injury and/or require hospitalization) on humans by month in British Columbia, Canada from 1960 to 2014 (n = 82).

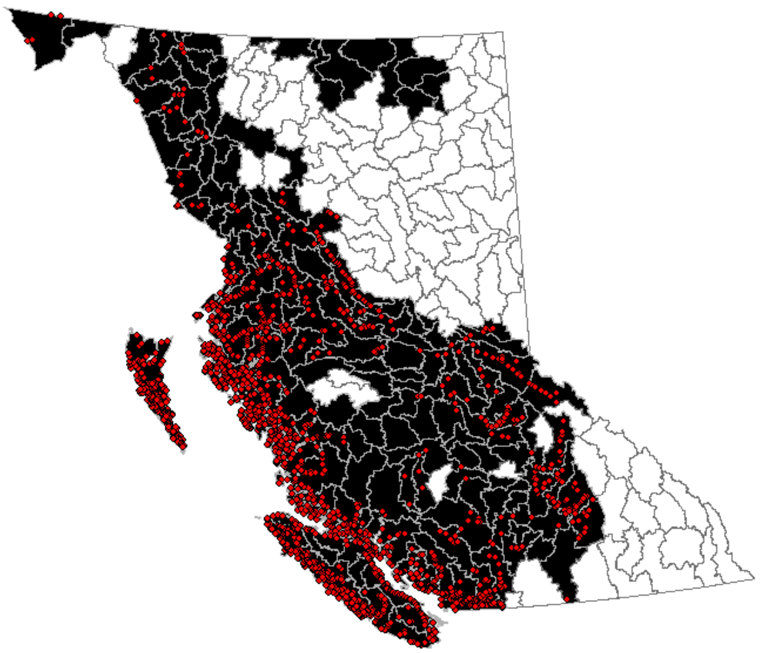

Supplementary Figure 7 Locations of spawning salmon counts within British Columbia, Canada. Polygons shown are watershed groups as defined by the BC watershed atlas [14,15]; spawning salmon (*Oncorhynchus* spp.) have been observed [16] in black watersheds, red dots are point locations where spawning salmon were enumerated [3]. Generated with ArcMap 10.2, www.esri.com.

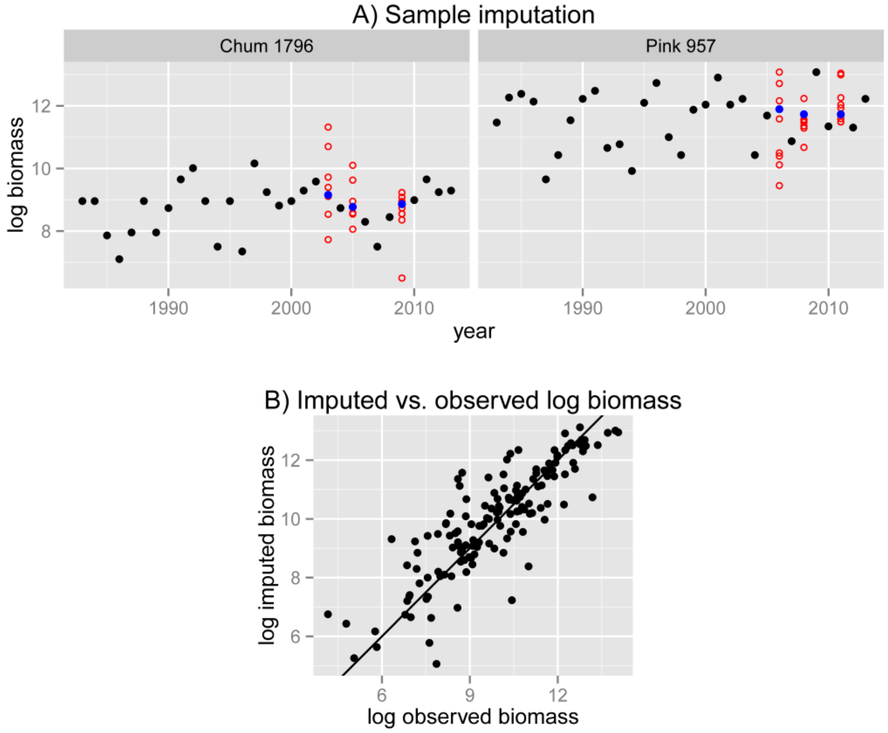

Supplementary Figure 8 A) Sample imputed time series for two salmon (*Oncorhynchus* spp.) count time-series in the Kwatna-Owikeno grizzly bear (*Ursus arctos horribilis)* population unit. Black dots represent log biomass from count data. Red dots represent eight draws from, and blue dots represent median value of, posterior distributions of values from a logistic Ricker-model used to impute biomass for years with missing count data. B) Comparison of median imputed versus observed log biomass, calculated by randomly removing and imputing one year of data from all complete time series (species counts in streams without any missing annual counts). Black line is the 1:1 line.

Supplementary Table 1 Model selection table of candidate models used to assess the effect of ecological variables on annual number of conflict-killed grizzly bears (*Ursus arctos horribilis)* in British Columbia, Canada, 1980-2013. Salmon represents yearly geometric mean of salmon biomass, Precip and Temp represent mean spring and summer temperature and total spring and summer precipitation, Hunt and Conflict represent the total number of hunter-killed and conflict-killed bears, respectively, in the previous three years, Grizzly pop represents estimated grizzly bear densities, Human pop represents estimated human population densities, Delta represents delta-AIC, and Weight represents model weight.

				**Annual variables**				**Spatial variables**						
	**Model**	**Intercept**	**Salmon**	**Precip**	**Temp**	**Previous conflict**	**Previous hunts**	**Precip**	**Temp**	**Grizzly pop**	**Human pop**	**Year**	**Delta**	**Weight**
Salmon Areas	8	-24.36	-0.42			0.29		-1.41	1.14	0.63	-0.08	0.02	0.00	0.32
	4	-24.36	-0.41					-1.43	1.14	0.64	-0.08	0.03	1.21	0.18
	5	-24.36	-0.43			0.28	-0.11	-1.40	1.13	0.63	-0.07	0.02	1.61	0.14
	6	-24.37	-0.42	0.25	0.57	0.31		-1.20	0.79	0.32	-0.13	0.02	2.54	0.09
	7	-24.36	-0.43				-0.14	-1.42	1.13	0.63	-0.06	0.03	2.56	0.09
	3	-24.36	-0.41	0.29	0.49			-1.32	0.83	0.36	-0.12	0.03	4.04	0.04
	1	-24.37	-0.43	0.21	0.58	0.30	-0.11	-1.15	0.77	0.30	-0.12	0.02	4.17	0.04
	15	-24.34				0.28		-1.36	1.12	0.57	-0.09	0.03	5.29	0.02
	2	-24.36	-0.43	0.24	0.51		-0.14	-1.25	0.81	0.35	-0.11	0.03	5.41	0.02
	16	-24.34						-1.38	1.12	0.58	-0.09	0.03	6.17	0.01
	14	-24.34				0.28	-0.06	-1.35	1.11	0.56	-0.08	0.02	7.21	0.01
	12	-24.35		0.11	0.62	0.30		-0.97	0.74	0.22	-0.15	0.03	7.75	0.01
	13	-24.34					-0.09	-1.37	1.11	0.57	-0.08	0.03	7.95	0.01
	9	-24.34		0.15	0.54			-1.10	0.79	0.27	-0.14	0.03	8.97	0.00
	10	-24.35		0.08	0.62	0.30	-0.07	-0.94	0.72	0.22	-0.14	0.03	9.66	0.00
	11	-24.34		0.12	0.55		-0.09	-1.05	0.77	0.26	-0.13	0.03	10.72	0.00
Full Region	7	-24.24				0.21		-0.76	0.55	0.98	1.22	0.03	0.00	0.41
	8	-24.24						-0.76	0.55	0.98	1.22	0.04	1.07	0.24
	6	-24.24				0.21	0.01	-0.76	0.56	0.98	1.22	0.03	2.03	0.15
	5	-24.24					0.00	-0.76	0.55	0.98	1.22	0.04	3.10	0.09
	4	-24.24		0.04	-0.04	0.21		-0.82	0.58	0.99	1.22	0.03	4.03	0.05
	1	-24.24		0.08	-0.08			-0.88	0.59	1.01	1.23	0.04	4.99	0.03
	2	-24.24		0.05	-0.04	0.21	0.01	-0.82	0.58	0.99	1.22	0.03	6.06	0.02
	3	-24.24		0.08	-0.08		0.00	-0.88	0.60	1.01	1.23	0.04	7.03	0.01

Supplementary Table 2 Candidate model set used to assess the effect of ecological variables on annual number of conflict-killed grizzly bears (*Ursus arctos horribilis)* in British Columbia, Canada, 1980-2013. Salmon represents annual geometric mean of salmon biomass, Precip and Temp represent mean spring and summer temperature and total spring and summer precipitation, Hunt and Conflict represent the total number of hunter-killed and conflict-killed bears, respectively, in the previous three years, Grizzly pop represents estimated grizzly bear densities, and Human pop represents estimated human population densities.

	**Model**	**Annual Variables**	**Spatial Variables**
Salmon Areas	1	salmon+temp+precip+hunt+conflict	temp+precip+human pop+grizzly pop
	2	salmon+temp+precip+hunt	temp+precip+human pop+grizzly pop
	3	salmon+temp+precip	temp+precip+human pop+grizzly pop
	4	salmon	temp+precip+human pop+grizzly pop
	5	salmon+hunt+conflict	temp+precip+human pop+grizzly pop
	6	salmon+temp+precip+conflict	temp+precip+human pop+grizzly pop
	7	salmon+hunt	temp+precip+human pop+grizzly pop
	8	salmon+conflict	temp+precip+human pop+grizzly pop
	9	temp+precip	temp+precip+human pop+grizzly pop
	10	temp+precip+hunt+conflict	temp+precip+human pop+grizzly pop
	11	temp+precip+hunt	temp+precip+human pop+grizzly pop
	12	temp+precip+conflict	temp+precip+human pop+grizzly pop
	13	hunt	temp+precip+human pop+grizzly pop
	14	hunt+conflict	temp+precip+human pop+grizzly pop
	15	conflict	temp+precip+human pop+grizzly pop
	16	no annual variables	temp+precip+human pop+grizzly pop
Full Region	1	temp+precip	temp+precip+human pop+grizzly pop+salmon present
	2	temp+precip+hunt+conflict	temp+precip+human pop+grizzly pop+salmon present
	3	temp+precip+hunt	temp+precip+human pop+grizzly pop+salmon present
	4	temp+precip+conflict	temp+precip+human pop+grizzly pop+salmon present
	5	hunt	temp+precip+human pop+grizzly pop+salmon present
	6	hunt+conflict	temp+precip+human pop+grizzly pop+salmon present
	7	conflict	temp+precip+human pop+grizzly pop+salmon present
	8	no annual variables	temp+precip+human pop+grizzly pop+salmon present

Supplementary References

1. Ministry of Forests, Lands and Natural Resource Operations. British Columbia grizzly bear population estimate for 2012. (2012).
2. British Columbia Ministry of Environment, Fish, Wildlife and Habitat Branch. Grizzly bear hunting: frequently asked questions. (2010).
3. FOC. *NuSEDS regional adult salmon escapement database 1950-2013*. (Fisheries and Oceans Canada, Pacific Biological Station, Nanaimo, BC, 2014).
4. Groot, C. & Margolis, L. *Pacific salmon life histories*. (UBC press, 1991).
5. Price, M. H. H., Darimont, C. T., Temple, N. F. & MacDuffee, S. M. Ghost runs: management and status assessment of Pacific salmon (*Oncorhynchus* spp.) returning to British Columbia's central and north coasts. *Can. J. Fish. Aquat. Sci.* **65,** 2712–2718 (2008).
6. Bryan, H. M., Darimont, C. T., Paquet, P. C., Wynne-Edwards, K. E. & Smits, J. E. G. Stress and reproductive hormones reflect inter-specific social and nutritional conditions mediated by resource availability in a bear–salmon system. *Conserv. Physiol.* **2,** (2014).
7. Delean, S., Brook, B. W. & Bradshaw, C. J. Ecologically realistic estimates of maximum population growth using informed Bayesian priors. *Methods Ecol. Evol.* **4,** 34–44 (2013).
8. Gelman, A. Prior distributions for variance parameters in hierarchical models (comment on article by Browne and Draper). *Bayesian Anal.* **1,** 515–534 (2006).
9. Gelman, A., Jakulin, A., Pittau, M. G. & Su, Y.-S. A weakly informative default prior distribution for logistic and other regression models. *Ann. Appl. Stat.* 1360–1383 (2008).
10. Carpenter, B. *et al.* Stan: a probabilistic programming language. *Journal of Statistical Software* (In press).
11. Homan, M. D. & Gelman, A. The no-u-turn sampler: adaptively setting path lengths in Hamiltonian Monte Carlo. *J Mach Learn Res* **15,** 1593–1623 (2014).

12. Stan Development Team. *Stan: a C++ library for probability and sampling, version 2.7.0*. (2015).
13. Quinn, T. P., Wetzel, L., Bishop, S., Overberg, K. & Rogers, D. E. Influence of breeding habitat on bear predation and age at maturity and sexual dimorphism of sockeye salmon populations. *Can. J. Zool.* **79,** 1782–1793 (2001).
14. British Columbia Ministry of Environment, Lands, and Parks, Fisheries Branch. *An introduction to the British Columbia watershed atlas*. (Spatial Vision Consulting, 1996).
15. Province of British Columbia. *User's guide to the British Columbia watershed/waterbody identifier system. Version 3.0.* (Ministry of Sustainable Resource Management, 2004).
16. BCGOV. *British Columbia historical fish distribution 50k spatial dataset*. (Ecosystems Branch, British Columbia Ministry of Environment, 2006).
